# Prevalence of smoking in patients with bipolar disorder, major depressive disorder and schizophrenia and their relationships with quality of life

**DOI:** 10.1038/s41598-017-07928-9

**Published:** 2017-08-16

**Authors:** Xiao-Hong Li, Feng-Rong An, Gabor S. Ungvari, Chee H. Ng, Helen F. K. Chiu, Ping-Ping Wu, Xin Jin, Yu-Tao Xiang

**Affiliations:** 1 The National Clinical Research Center for Mental Disorders & Beijing Key Laboratory of Mental Disorders, Beijing Anding Hospital, Capital Medical University, Beijing, China; 2 Unit of Psychiatry, Faculty of Health Sciences, University of Macau, Macao, SAR China; 3 The University of Notre Dame Australia/Marian Centre, Perth, Australia; 4 School of Psychiatry & Clinical Neurosciences, University of Western Australia, Perth, Australia; 5 Department of Psychiatry, University of Melbourne, Melbourne, Victoria Australia; 6 Department of Psychiatry, Chinese University of Hong Kong, Hong Kong, SAR China

## Abstract

Few studies have compared the prevalence of smoking between patients with bipolar disorder, major depressive disorder (MDD) and schizophrenia. This study examined the prevalence of smoking and its relationships with demographic and clinical characteristics, and quality of life (QOL) in patients with these psychiatric disorders. A total of 1,102 inpatients were consecutively screened. Psychopathology and QOL were measured with standardized instruments. The prevalence of current smoking in the whole sample was 16.7%; 17.5% in bipolar disorder, 10.6% in MDD and 18.5% in schizophrenia. The rates of smoking in bipolar disorder (p = 0.004, OR = 2.5, 95%CI: 1.3–4.7) and schizophrenia (p = 0.03, OR = 2.0, 95%CI: 1.06–3.8) were significantly higher than in MDD, while no difference was found between bipolar disorder and schizophrenia. Smokers had a higher mental QOL than non-smokers (p = 0.007) in MDD, but no difference was found in the other two groups. Male gender, living alone, higher personal income, older age of onset, health insurance coverage, and first episode was significantly associated with smoking in one or more diagnostic groups. Smoking appears more common in bipolar disorder and schizophrenia than in MDD in China. The figures in all disorders were lower than that reported in most of other countries.

## Introduction

Smoking is a major public health challenge worldwide, killing around six million people per year^1^. Evidence has shown that patients with major psychiatric disorders are prone to smoking^2–10^. A meta-analysis found prevalence of smoking in schizophrenia was 62%, which is 5.3 times higher than in the general population^4^. In Western studies, the prevalence of smoking in other psychiatric disorders seems to be lower than in schizophrenia, e.g. 31.2–66% in bipolar disorder^11–14^, and 34–60% in depression^12, 13, 15^, but these rates are still much higher than in the general population. Smoking could lead to smoking-related medical conditions, increased risk of premature death and huge economic burden^16, 17^. Therefore, understanding smoking patterns in major psychiatric disorders is important to develop effective interventions for smoking cessation and reduction of harmful outcomes.

Patients with major psychiatric disorders are vulnerable to smoking but the vulnerability is also influenced by the sociocultural context^18–21^. For example, de Leon *et al*.^4^ found lower smoking rates in psychiatric patients than in the general population in Japan and Columbia. Thus, findings on smoking reported from Western settings may not be applicable to other cultural contexts. Further, only a few Western studies have directly compared smoking rates across different major psychiatric disorders^3, 22^.

China has a population of 1.4 billion and the 1-month prevalence of any psychiatric disorder was estimated to be 17·5%^23^. To the best of our knowledge, no previous study has compared the smoking rates directly between different major psychiatric disorders in China. This study examined the prevalence of smoking in patients with bipolar disorder, major depressive disorder (MDD) and schizophrenia, and explored the relationships between smoking and demographic and clinical characteristics, and quality of life (QOL) in a large representative sample. Based on previous findings^3, 22^, we expected that the prevalence of smoking would be highest in schizophrenia, followed by bipolar disorder and MDD. In addition, smoking rates in each of these disorders is expected to be higher than in the general population in China.

## Methods

### Participants and study site

The study, conducted from February to August 2013, was part of a larger research project that examined the quality of psychiatric service models^24, 25^ at the China’s National Clinical Research Center for Mental Disorders. This center has 800 acute psychiatric beds and receives more than 1,500 outpatient visits daily, and provides mental health services for over 20 million people in northern China. Patients who were admitted to the hospital during the study period were consecutively screened for eligibility based on the following study entry criteria: (1) diagnosis of schizophrenia or other psychotic disorders (schizophrenia thereafter), bipolar disorder or major depressive disorder (MDD) according to DSM-IV criteria; if there were multiple diagnoses, only the principal diagnosis was recorded; (2) age 18 years or older; (3) ability to understand the contents of the protocol; (4) willingness to provide written informed consent.

### Instruments and evaluation

Demographic and clinical data were collected by a review of medical records using a standardized form designed for this study and confirmed with a clinical interview with the patient and family members if available.

Smoking-related variables in this study were defined as follows: lifetime smoking referred to smoking at least 1 cigarette daily for at least one month at some time in the past^7, 26^; and current smoking was defined as smoking at least 1 cigarette daily during the past month^7^ prior to the current admission. Age of smoking onset was also recorded. In the case of any discrepancy between patients and their families, a family meeting was conducted to reach a consensus.

The validated Chinese version of the Symptom Checklist-90 (SCL-90) was used to measure psychiatric symptoms; its 90 items covers nine areas: somatization, obsession-compulsion, interpersonal sensitivity, depression, anxiety, hostility, phobic anxiety, paranoid ideation, and psychoticism. Each area is rated on a 5-point scale ranging from 0 (absence of the symptom) to 4 (maximum intensity score for the symptom)^27^. Only the total score was used in this study. QOL was assessed with the validated Chinese version of the Medical Outcomes Study Short Form-12 (SF-12)^28, 29^. This is a multidimensional generic instrument with 12 items covering physical and mental component scores. A higher score on SF-12 represents better QOL.

Two psychiatric nurses with over 5 years’ experience in clinical research interviewed patients within 48 hours after admission. The study protocol was approved by the Biomedical Ethics Board of Beijing Anding Hospital, China. The protocol including the methods was performed in accordance with the Declaration of Helsinki and the relevant ethical guidelines and regulations in China. Informed consents were obtained from all patients. No identifying information/images were involved in this study.

### Statistical analysis

All data were analyzed using SPSS 21.0 for Windows. Basic socio-demographic and clinical characteristics between different diagnostic groups and between smokers and non-smokers were compared using independent sample t-test, Kruskal-Wallis H test, and chi-square test, as appropriate. QOL was compared between smokers and non-smokers in each diagnostic group with analysis of covariance (ANCOVA) after controlling for the potentially confounding effects of variables that were significantly different in univariate analyses. Multiple logistic regression with the “Enter” method was performed to compare the prevalence of smoking between the three diagnostic groups after controlling for variables that differed significantly in univariate analyses. Furthermore, the independent demographic and clinical correlates of smoking were examined using multiple logistic regression with the “Enter” method separately in each diagnostic group, with smoking as the dependent variable and the socio-demographic and clinical variables that were significantly different in univariate analyses as independent variables. As the smoking rate is significantly higher in male patients in China^5, 6^, the independent demographic and clinical correlates of smoking in male patients were examined using multiple logistic regression with the “Enter” method. The level of significance was set at 0.05 (two-tailed).

## Results

Of the 1,475 patients admitted during the study period, 1,102 met the study criteria and participated in the study. The prevalence of lifetime smoking in the whole sample was 19.6%; 20.9% in bipolar disorder, 13.2% in MDD and 20.9% in schizophrenia, while the corresponding figures of current smoking were 16.7% (male: 38.4%; female: 4.3%), 17.5% (male: 41.2%; female: 6.3%), 10.6% (male: 30.4%; female: 2.3%) and 18.5% (male: 38.6%; female: 2.8%). The prevalence of current smoking was highest in the schizophrenia group, followed by the bipolar disorder and MDD groups. There was no significant difference between the three diagnostic groups in the proportion of patients starting smoking prior to the illness, daily amount of current smoking and age of smoking onset.

There were significant group differences in terms of age, male gender, education level, marital status, personal income, major medical conditions, family history of psychiatric disorders, age of onset, first episode of the illness, length of illness and number of admissions (Table 1). After controlling for the variates that significantly differed in univariate analyses, the prevalence of smoking in bipolar disorder (p = 0.004, OR = 2.5, 95%CI: 1.3–4.7) and schizophrenia (p = 0.03, OR = 2.0, 95%CI: 1.06–3.8) were significantly higher than in MDD, but no significant difference between bipolar disorder and schizophrenia groups was found (p = 0.33).

**Table 1:** Basic socio-demographic and clinical characteristics of the study sample.

	Whole sample (n = 1102)		Schizophrenia (n = 449)		Bipolar disorder (n = 464)		Major depressive disorders (n = 189)		Statistics
	N	%	N	%	N	%	N	%	***P*** **value**
Male	401	36.4	197	43.9	148	31.9	56	29.6	<**0.001**
Married/cohabitating	493	44.7	155	34.5	207	44.6	131	69.3	<**0.001**
First episode	264	24.0	89	19.8	113	24.4	62	32.8	**0.002**
Living alone	79	7.2	34	7.6	33	7.1	12	6.3	0.85
Personal income (>3000 RMB)	228	20.7	64	14.3	118	25.4	46	24.3	<**0.001**
Health insurance	668	60.6	279	62.1	268	57.8	121	64.0	0.23
Major medical conditions	204	18.5	77	17.1	72	15.5	55	29.1	<**0.001**
Family history of psychiatric disorders	341	30.9	151	33.6	149	32.1	41	21.7	**0.009**
Lifetime smoking	216	19.6	94	20.9	97	20.9	25	13.2	0.053
Current smoking	194	16.7	83	18.5	81	17.5	20	10.6	**0.04**
Started smoking prior to illness	197	17.9	86	19.2	87	18.8	24	12.7	0.1
	**Mean**	**SD**	**Mean**	**SD**	**Mean**	**SD**	**Mean**	**SD**	***P*** **value**
Age (years)	36.0	14.3	34.5	12.8	33.5	13.4	45.4	15.9	<**0.001**
Education (years)	11.9	3.3	11.9	3.1	12.2	3.2	11.3	3.8	**0.03**
Age of onset (years)	27.6	12.4	24.4	9.8	26.1	10.8	38.8	15.4	<**0.001**
Length of illness (years)	8.4	9.5	10.1	10.1	7.4	9.03	6.7	8.9	<**0.001**
Number of admissions	2.6	2.2	2.9	2.5	2.5	2.1	1.9	1.3	<**0.001**
SCL90 total	162.7	88.9	164.1	102.9	158.3	81.2	170.5	68.5	0.07
Daily smoking amount (no. of cigarettes)	12.0	4.7	12.0	5.7	12.1	4.3	11.8	3.0	0.5
Age at first cigarette use	18.3	3.7	18.2	4.0	18.4	3.6	18.4	3.1	0.3

Bolded values: <0.05; Kruskal-Wallis H test was used to compare continuous variables; 1 USD = 6 RMB; SCL-90 = Symptom Checklist-90; SF-12 = Medical Outcomes Study Short Form. The one-sample Kolmogorov-Smirnov test was used to check the normality of distribution for continuous variables.

Table 2 compares demographic and clinical characteristics between smoking and non-smoking groups by diagnoses. In bipolar disorder, after controlling for male gender, living alone, personal income, having health insurance, age and age of onset, there was no significant difference between the smoking and non-smoking groups in physical (F_(1,456)_ = 0.1, P = 0.67) and mental QOL (F_(1, 456)_ = 0.002, P = 0.96). In MDD, after controlling for male gender, age and age of onset, there was no significant difference between the smoking and non-smoking groups in physical (F_(1,184)_ = 0.3, P = 0.57) QOL, but smokers had a higher mental QOL than non-smokers (F_(1,184)_ = 7.5, P = 0.007). In schizophrenia, after controlling for male gender, first episode, education level and the SCL-90 total score, there was no significant difference between the smoking and non-smoking groups in physical (F_(1,400)_ = 1.6, P = 0.20) and mental QOL (F_(1,400)_ = 0.001, P = 0.98).

**Table 2:** Comparisons of demographic and clinical characteristics between smoking and non-smoking groups by diagnoses.

	Schizophrenia (n = 449)				Bipolar disorder (n = 464)				Major depressive disorders (n = 189)			
	Non-Smoking (n = 366)		Smoking (n = 83)		Non-Smoking (n = 383)		Smoking (n = 81)		Non-Smoking (n = 169)		Smoking (n = 20)	
	N	%	N	%	N	%	N	%	N	%	N	%
Male gender	121	33.1	76	91.6**	87	22.7	61	75.3**	39	23.1	17	85.0**
Married/cohabitating	124	33.9	31	37.3	167	43.6	40	49.4	118	69.8	13	65.0
First episode	66	18.0	23	27.7*	97	25.3	16	19.8	59	34.9	3	15.0
Living alone	24	6.6	10	12.0	19	5.0	14	17.3*	11	6.5	1	5.0
Personal income (>3000 RMB)	48	13.1	16	19.3	86	22.5	32	39.5*	40	23.7	6	30.0
Health insurance	223	60.9	56	67.5	201	52.5	67	82.7*	109	64.5	12	60.0
Major medical conditions	62	16.9	15	18.1	56	14.6	16	19.8	50	29.6	5	25.0
Family history of psychiatric disorders	123	33.6	28	33.7	119	31.1	30	37.0	36	21.3	5	25.0
	**Mean**	**SD**	**Mean**	**SD**	**Mean**	**SD**	**Mean**	**SD**	**Mean**	**SD**	**Mean**	**SD**
Age (years)	34.2	13.0	36.1	11.8	32.8	13.6	37.1**	11.6	46.3	15.9	38.1*	14.7
Education (years)	12.1	3.1	11.2*	3.1	12.3	3.2	11.6	3.0	11.2	3.9	11.7	2.8
Age of onset (years)	24.1	10.0	25.6	8.6	25.6	10.9	28.8*	9.6	39.6	15.4	31.2*	14.1
Length of illness (years)	10.3	10.3	9.6	9.1	7.4	8.9	7.4	9.4	6.7	9.1	6.5	6.6
Number of admissions	2.8	2.4	3.4	3.0	2.4	2.0	2.6	2.4	1.9	1.3	1.7	0.9
SCL90 total	158.1	57.8	188.9*	200.5	155.1	84.4	173.2	62.2	168.6	68.7	185.6	67.1
SF-12 Physical	57.5	15.5	54.1	16.5	58.1	17.3	57.8	16.6	62.1	16.5	65.4	13.2
SF-12 Mental	42.9	18.4	43.6	19.4	42.3	18.9	42.8	18.9	37.7	19.8	43.4	14.0

*p < 0.05; **p < 0.01; SCL-90 = Symptom Checklist-90; SF-12 = Medical Outcomes Study Short Form.

Table 3 shows the demographic and clinical correlates independently associated with smoking by diagnoses. Smoking was independently associated with male gender and older age of onset in schizophrenia. Smoking was positively associated with male gender, living alone, higher personal income and health insurance, while negatively associated with first episode of illness and education level in bipolar disorder. Smoking was positively associated with male gender, but negatively associated with age of onset in MDD. In the pooled sample of male patients, smoking was positively associated with living alone and the number of admissions (Table 4).

**Table 3:** Demographic and clinical correlates independently associated with smoking by diagnoses.

	Schizophrenia			Bipolar disorder			Major depressive disorders		
	*P* value	OR	95% C.I.	*P* value	OR	95% C.I.	*P* value	OR	95% C.I.
Male gender	**<0.001**	31.0	12.0–80.2	**<0.001**	16.3	7.9–33.3	**<0.001**	32.2	6.0–171.7
Married/cohabitating	0.31	1.5	0.6–3.3	0.93	0.9	0.4–2.1	0.18	3.2	0.5–18.3
First episode	0.12	1.7	0.8–3.6	**0.01**	0.3	0.1–0.7	0.11	0.2	0.03–1.4
Living alone	0.06	2.6	0.9–7.4	**0.001**	5.4	1.9–15.1	0.99	<0.001	—
Personal income (>3000 RMB)	0.40	1.4	0.6–3.3	**0.03**	2.2	1.07–4.7	0.42	1.7	0.4–7.4
Health insurance	0.90	1.0	0.5–2.0	**<0.001**	4.8	2.2–10.7	0.24	2.5	0.5–12.7
Major medical conditions	0.96	0.9	0.4–2.3	0.10	0.4	0.1–1.1	0.32	2.4	0.4–14.0
Family history of psychiatric disorders	0.74	1.1	0.5–2.1	0.97	0.9	0.4–1.9	0.93	0.9	0.2–4.0
Education (years)	0.31	0.9	0.8–1.0	**0.01**	0.8	0.7–0.9	0.7	0.9	0.7–1.1
Age of onset (years)	**0.01**	1.04	1.008–1.08	0.21	1.0	0.9–1.0	**0.03**	0.9	0.8–1.0
Length of illness (years)	0.89	1.0	0.9–1.04	0.90	1.0	0.9–1.0	0.14	0.9	0.9–1.0
Number of admissions	0.06	1.1	0.9–1.2	0.45	1.0	0.9–1.2	0.49	0.7	0.4–1.5
SCL-90 total	0.27	1.0	0.9–1.0	0.82	1.0	0.9–1.0	0.5	1.003	0.9–1.0

Bolded values: <0.05; SCL-90 = Symptom Checklist-90; SF-12 = Medical Outcomes Study Short Form; OR = Odds ratio; Due to collinearity between age and age of onset in schizophrenia, between age and age of onset and length of illness in bipolar disorder and between age and age of onset and length of illness in major depressive disorders, age was not entered as independent variable in the above multiple logistic regression analyses.

**Table 4:** Comparisons of demographic and clinical characteristics between smoking and non-smoking in male patients.

	Male patients (n = 401)		
	*P* value	OR	95% C.I.
Married/cohabitating	0.13	1.5	0.8–2.8
First episode	0.19	0.6	0.3–1.2
Living alone	**0.01**	3.0	1.2–7.2
Personal income (>3000 RMB)	0.09	1.5	0.9–2.7
Health insurance	0.06	1.5	0.9–2.6
Major medical conditions	0.41	0.7	0.3–1.4
Family history of psychiatric disorders	0.31	0.7	0.4–1.2
Diagnosis			
Schizophrenia	—	1.0	—
Bipolar disorder	0.10	1.9	0.8–4.1
Major depressive disorders	0.07	2.0	0.9–4.3
Education (years)	0.43	0.9	0.8–1.0
Age of onset (years)	0.16	1.0	0.9–1.0
Length of illness (years)	0.93	1.0	0.9–1.0
Number of admissions	**0.03**	1.1	1.0–1.2
SCL-90 total	0.22	1.0	0.9–1.0

Bolded values: <0.05; SCL-90 = Symptom Checklist-90; OR = Odds ratio; Due to collinearity between age and age of onset and length of illness, age was not entered as independent variable in the above multiple logistic regression analysis.

## Discussion

To our best knowledge, this was the first study that compared the prevalence of smoking between patients with schizophrenia, bipolar disorder and MDD in China. The prevalence of current smoking was 16.7% in the whole sample; 17.5% in bipolar disorder, 10.6% in MDD and 18.5% in schizophrenia. The prevalence in the whole sample was significantly lower than previous findings (61.3–73.5%) reported in both Chinese^30–33^ and Western psychiatric patient samples^34^, and even lower than the smoking rate (28.1%) in adult Chinese general population^35^. Lasser *et al*.^7^ reported that the prevalence of current smoking in persons with any lifetime psychiatric disorders was 34.8%, which is much higher than our results. Differences in types of psychiatric disorders, study settings, sample size, demographic and clinical characteristics, and sampling method may contribute to the discrepancy in the findings between studies. For example, there are usually smoking restrictions in inpatient settings^19^, which may contribute to the low smoking rate. Due to the gender differences, gender ratio of population samples is also a contributing factor to differences in smoking rate^20^. In contrast to the high ratio of males (93.8–100%) in previous studies^30–33^, male patients only accounted for 36.4% of the whole sample in this study.

There are several reasons for the association between severe psychiatric disorders and smoking. There is preliminary evidence that the variants in the 15q25 gene cluster may be a contributing factor to smoking in patients with schizophrenia and bipolar disorders^31, 36^. In addition, the abnormality of nicotine receptor (a7) may be another genetic factor making schizophrenia patients vulnerable to smoking^37, 38^. Furthermore, psychotic symptoms and antipsychotic-induced side effects could be mitigated through nicotinic receptors in the mesolimbic and nigrostriatal systems^39, 40^.

As expected, the prevalence of smoking was highest in schizophrenia, followed by bipolar disorder and MDD. However, considering that smoking rate is largely influenced by gender ratio^20^, we also examined the effect of gender in each diagnostic group. Surprisingly, smoking rate was highest in bipolar disorder (41.2%), followed by schizophrenia (38.6%) and MDD (30.4%) in males, with a similar pattern found in females: highest in bipolar disorder (6.3%), followed by schizophrenia (2.8%) and MDD (2.3%). This is inconsistent with previous Western findings that smoking is most common in schizophrenia^3, 39, 41–44^. In addition, smoking rates across each of the three disorders in this study were significantly lower than previous findings; e.g., Diaz *et al*.^22^ found smoking rate was 66%, 57% and 74% in patients with bipolar disorder, MDD and schizophrenia, respectively in a cohort of 424 psychiatric patients. Of note, the age of schizophrenia patients in our study was relatively young (34.5 years) and 19.8% had first-episode disorder. It is possible that local Chinese culture may have variable influence on smoking behavior in first-episode and chronic patients^20, 45^. In multivariate analyses no significant difference between bipolar disorder and schizophrenia in smoking rates was found, in line with the notion that both schizophrenia and bipolar disorder patients are vulnerable to tobacco use^3, 4, 12, 46^. Smoking rate was lowest in MDD in this study, which supports previous studies that found no strong cause-effect relationship between depression and smoking^47–49^.

Similar to previous studies^14, 50, 51^, male gender was a major risk factor for smoking in all diagnostic groups in this study. Further, smokers with bipolar disorder were more likely to have lower education and live alone compared to non-smokers, which is consistent with earlier findings^20, 52^. The cost of cigarette has been associated with smoking rate^53^. Patients with a higher income and health insurance coverage who could afford buying cigarettes were more likely to smoke. This may partly explain the higher rate of smoking in bipolar patients who had a relatively higher percentage of health insurance than the other two groups. As better education brings greater awareness of the harmful effects of smoking, patients with higher education are less likely to smoke^26^. In contrast, those living alone with poor family support may be more inclined to smoke as a behavioral filler^54^. Similar to other studies^20, 55^, older age of onset was positively associated with smoking in schizophrenia, but negatively associated with smoking in MDD in this study^9, 56^.

Demographic correlates of smoking were evident in patients with bipolar disorders. Patients living alone experience more negative feelings due to lack of family support, thus were more likely to smoke to relieve loneliness and perhaps boredom^14, 54^. Similar to previous findings^14^, smokers also had lower income. We have no explanation why smoking was associated with first episode illness, higher income and health insurance in this group only. These findings need to be confirmed in future studies.

More frequent admissions were positively associated with smoking in male patients. It is likely that patients with more frequent relapses and hospitalizations had more severe illness and higher antipsychotic dosages that are associated with increased rate of smoking. Despite the negative impact of smoking on health, patients with MDD who smoked had a higher QOL. Self-medication as a coping strategy may be a factor since smoking may alleviate cognitive deficits, lessen medication side effects, improve attention and concentration and relieve depressive and anxiety symptoms^7, 57–62^. All these aspects of smoking could improve QOL. However, other studies did not support the “self-medication” hypothesis^8, 34, 63^.

There are several limitations to this study. First, due to the cross-sectional study design, causality between smoking and other variables could not be determined. Second, some factors which influence smoking, such as lifestyle and social support, were not examined. Third, there is no substance dependence ward in this hospital, thus the association between comorbid substance dependence and smoking could not be examined. The study design focused only on the frequency of cigarettes use, and specific nicotine dependence was not examined. Fourth, smoking was self-reported, thus the possibility of recall bias cannot be excluded. Fifth, the study was only conducted in one large tertiary referral hospital hence the results cannot be generalized to other settings.

## Conclusions

The prevalence of current smoking in patients with schizophrenia and bipolar disorders were significantly higher than in MDD in China. However, the figures for all three disorders were considerably lower than those reported in Western countries. The influence of psychopathological and socio-cultural factors on smoking warrants further investigations.
